# Potential Role of Humoral IL-6 Cytokine in Mediating Pro-Inflammatory Endothelial Cell Response in Amyotrophic Lateral Sclerosis

**DOI:** 10.3390/ijms19020423

**Published:** 2018-01-31

**Authors:** Svitlana Garbuzova-Davis, Jared Ehrhart, Paul R. Sanberg, Cesario V. Borlongan

**Affiliations:** 1 Center of Excellence for Aging & Brain Repair, Morsani College of Medicine, University of South Florida, 12901 Bruce B. Downs Blvd., MDC 78, Tampa, FL 33612, USA; jehrhar1@health.usf.edu (J.E.); psanberg@usf.edu (P.R.S.); cborlong@health.usf.edu (C.V.B.); 2 Department of Neurosurgery and Brain Repair, Morsani College of Medicine, University of South Florida, Tampa, FL 33612, USA; 3 Department of Molecular Pharmacology and Physiology, Morsani College of Medicine, University of South Florida, Tampa, FL 33612, USA; 4 Department of Pathology and Cell Biology, Morsani College of Medicine, University of South Florida, Tampa, FL 33612, USA; 5 Department of Psychiatry, Morsani College of Medicine, University of South Florida, Tampa, FL 33612, USA

**Keywords:** ALS, IL-6 cytokine, endothelial cells, inflammation

## Abstract

Amyotrophic lateral sclerosis (ALS) is a multifactorial disease with limited therapeutic options. Numerous intrinsic and extrinsic factors are involved in ALS motor neuron degeneration. One possible effector accelerating motor neuron death in ALS is damage to the blood-Central Nervous System barrier (B-CNS-B), mainly due to endothelial cell (EC) degeneration. Although mechanisms of EC damage in ALS are still unknown, vascular impairment may be initiated by various humoral inflammatory factors and other mediators. Systemic IL-6-mediated inflammation is a possible early extrinsic effector leading to the EC death causing central nervous system (CNS) barrier damage. In this review, we discuss the potential role of humoral factors in triggering EC alterations in ALS. A specific focus was on humoral IL-6 cytokine mediating EC inflammation via the trans-signaling pathway. Our preliminary in vitro studies demonstrated a proof of principle that short term exposure of human bone marrow endothelial cells to plasma from ALS patient leads to cell morphological changes, significantly upregulated IL-6R immunoexpression, and pro-inflammatory cell response. Our in-depth understanding of specific molecular mechanisms of this humoral cytokine in EC degeneration may facilitate an endothelial-IL-6-targeting therapy for restoring cell homeostasis and eventually reestablishing B-CNS-B integrity in ALS.

## 1. Introduction

Amyotrophic lateral sclerosis (ALS) is a neurodegenerative disease affecting motor neurons in the brain and spinal cord. Progression of this disease leads to paralysis and death of the patient, usually within five years of diagnosis [1,2,3]. About 90–95% of ALS cases are sporadic (SALS) while the remaining cases are genetically linked or familial (FALS). Men have a higher incidence of ALS than women and peak ages for initial disease symptoms are 58–63 years for SALS and 47–52 years for FALS [4]. In FALS cases, various mutations in genes coding for Cu/Zn superoxide dismutase 1 (*SOD1*), *TARDBP* (*TDP-43*), *FUS/TLS*, *ANG*, and *C90RF72* have been identified (reviewed in [5,6,7,8,9]). Although a mutation in the *C90RF72* gene was mainly associated with FALS, this gene mutation has also been found in some SALS cases [10,11]. The clinical presentation and underlying pathology of SALS and FALS are similar. Initially, muscle weakness and twitching or cramping of legs or arms appear in ALS patients. As the disease progresses, muscle atrophy, loss of motor control, and decreased range or stamina are observed. Also, dysarthria, dysphagia, fasciculations, and hyperreflexia are common features of ALS, depending upon the upper and/or lower motor neuron dysfunction. At the end disease stage, muscular paralysis and death occur due to respiratory failure. These clinical disease manifestations have been discussed in detail (reviewed in [12,13,14,15,16,17]). However, regardless of the part of the body first affected by the disease, muscle weakness and atrophy spread to other parts of the body as the disease progresses. Developing specific tools for evaluation of clinical symptoms in ALS patients is very important not only for early diagnosis but also for measuring disease progression, i.e., monitoring swallowing or dysphagia [18,19]. In spite of intensive research on ALS pathogenesis, numerous intrinsic and extrinsic factors in motor neuron death (reviewed in [15,20,21,22,23,24]) limit therapeutic options. The only USA Food and Drug Administration approved drugs for ALS are riluzole [25] and the recently approved edaravone (Radicava^®^, Mitsubishi Tanabe Pharma Corporation, Osaka, Japan) [26]. Riluzole acts to block the release of excitotoxic glutamate [27] while edaravone has anti-oxidant properties [26].

One possible effector accelerating motor neuron death in ALS is damage to the blood-CNS barrier [28], which separates the CNS tissue from detrimental factors in the systemic circulation. Impairment of the blood-brain barrier (BBB) and blood-spinal cord barrier (BSCB), (collectively, the blood-CNS barrier, B-CNS-B), has been shown in a mouse model of disease and in ALS patients [29,30,31,32,33,34,35,36,37,38]. Our [29,30,31,32] and other [33,34,35,36,37,38] studies demonstrated degeneration of microvessel endothelial cells (EC) and perivascular astrocyte end-feet processes, impairment of the endothelial transport system, and dysfunction of tight junction proteins, deficiencies associated with compromised barrier integrity in the brain and spinal cord, which lead to blood vessel leakage in motor neuron areas. Thus, vascular damage may be an early ALS pathological event [33,34,35]. These and other recent discoveries may identify ALS as a neurovascular disease [32,39,40]. However, mechanism(s) of EC degeneration in ALS is still unknown. 

Since the CNS endothelium is a specialized barrier isolating the blood compartment from brain/spinal cord parenchyma, initial microvascular EC damage may be due to blood-derived inflammatory and other mediators in ALS. Elevated systemic levels of inflammatory cytokines such as tumor necrosis factor-alpha (TNF-α), interleukin (IL)-6, IL-8, interferon-beta (IFN-β), and other interleukins have been identified in ALS [41,42,43,44,45]. Moreover, these peripheral biomarkers not only indicate ongoing inflammatory processes in ALS patients, but also may be used to distinguish ALS patients from patients with other neurological diseases [7,46] and even to predict ALS prognosis [47]. Also, increased cytokine levels detected in blood from ALS patients could be important mediators of the peripheral inflammatory response, either by promoting neuroprotection or accelerating disease progression. Notably, IL-8 is not only an inflammatory cytokine with chemoattractive activity predominantly for neutrophils, but is also a potent angiogenic factor [48]. However, our particular interest is mechanisms of IL-6 actions since this bi-functional cytokine can serve as an anti- or pro-inflammatory mediator [49,50,51,52]. Recognizing IL-6′s dual actions, it will be critical to monitor humoral expression levels of this cytokine during disease progression in ALS patients. Peripheral IL-6 upregulation likely corresponds to an inflammatory cell response exacerbating EC damage. This correspondence identifies the IL-6 cytokine as a potential target for a future modulating therapy in ALS. 

In this review, we discuss the potential role of humoral factors for triggering EC alterations in ALS. A specific focus was on humoral IL-6 cytokine as a latent early extrinsic inflammatory effector leading to EC damage. Our understanding of specific molecular mechanisms of this cytokine in EC degeneration may foster development of targeted therapies for restoring cell homeostasis and eventually restoring B-CNS-B integrity in ALS. 

## 2. Humoral Effectors in ALS Patients

B-CNS-B impairment has been detected in ALS, suggesting that barrier breakdown is a significant contributor to disease progression [14,15,16,17,18,19,20,21,22,23]. Besides determining systemic biomarkers associated with ALS, it is also important to evaluate detrimental effect(s) of humoral factors on endothelium homeostasis since ECs comprise the first lining of cells separating the blood compartment from CNS tissues [53,54]. 

Recently, a meta-analysis [55] provided a systematic review of 25 publications regarding blood inflammatory cytokines in ALS patients vs. control subjects. Results showed that the levels of TNF-α, TNF receptor 1, IL-6, IL-1β, IL-8, and vascular endothelial growth factor (VEGF) were significantly higher in ALS patients compared to controls, suggesting that these peripheral inflammatory cytokines might be biomarkers for ALS. Potential diagnostic and/or prognostic biomarkers, particularly in systemic compartment, of disease have been intensively investigated over the last decade. Robelin et al. [56] comprehensively reviewed humoral biomarkers at the molecular and cellular levels relevant to major pathogenic mechanisms contributing to motor neuron degeneration in ALS, such as excitotoxicity, oxidative stress, inflammation, metabolic dysfunction, apoptosis, and axonopathy. However, the authors [56] noted that since no proposed systemic biomarkers have yet translated to a clinical setting, several obstacles must be addressed. The authors proposed that it would be “more appropriate to identify panels of biomarkers, rather than focusing on a single gene, protein, or metabolite”. In agreement with the authors’ remark, we also believe that specification of biomarkers is challenging due to systemic changes during disease progression. Additional factors, such as ALS type (familial or sporadic), anatomical onset of motor neuron impairment (upper, lower, or bulbar), and even age of initial symptoms including gender bias may influence variability of investigated biomarkers. In our opinion, cellular sources of various proteins, which might modify peripheral blood content, should also be taken into account. 

Additionally, peripheral immune cells contribute to ALS pathogenesis, potentially reflecting an adaptive immune/inflammatory system response (reviewed in [57,58,59]). In SALS patients, increased CD4+ and decreased CD8+ cell levels as well as significantly reduced CD4+CD25+ regulatory T cells (Treg) and CD14+ monocytes were noted in blood [60]. Decreased numbers of Treg lymphocytes have been shown to correlate with rapid disease progression in patients, potentially indicating immune dysfunction in later ALS stages [61]. In contrast, another study [62] showed significant increases of CD8 cytotoxic T cells and natural killer (NK) cells in blood of ALS patients. Additionally, activated macrophages were observed in blood of SALS patients, which persisted throughout the course of disease [63]. The authors also reported that expression of HLA-DR on CD14+ monocytes was related to the rate of disease progression, suggesting a direct relationship between humoral macrophage activation and ALS disease stage. Also, counts of CD16+ peripheral monocytes [64] and neutrophil/lymphocyte ratio [65] were elevated in ALS patients. Relatively recently, a significant increase of neutrophils and decreased CD4+ T cells and CD16− monocytes were shown in ALS patients’ blood, resulting in an increased ratio of neutrophils to CD16− monocytes (N:M ratio) [66]. The authors suggest that reduction of CD4+ lymphocytes and CD16− monocytes reflects extravasation of these cells from the blood into the tissues and that the N:M ratio might be a helpful marker of disease progression. Interestingly, the study showed changes in T cell and monocyte cell populations including IL-6 production between identical female twins, one of which developed ALS and the other did not [67]. Results demonstrated more abundant serum IL-6 and TNF-α productions by macrophages in addition to the presence of CD8+ effector T cells in the ALS-twin vs. the non-ALS twin, leading to the conclusion that high expression of these toxic cytokines on infiltrating macrophages into ALS tissues might contribute to increased inflammatory response. 

Thus, cross-linking between the peripheral immune and inflammatory cell responses in ALS likely indicates the complexity of a dynamic immune/inflammatory system response. The complicated interactions would be not only dependent on the current disease stage but also on the humoral content of specific biomarkers. Since inflammation reflects a cascade of processes underlying particular cellular system responses largely controlled by actions of different mediators released under inflammatory conditions, monitoring the status of inflammatory mediators in the same patient during disease progression might be a more useful strategy.

In partial support of this suggestion, we demonstrated changes in SALS patients’ humoral factors during disease progression [45]. Cytokines and other factors such as nitrite and glutathione (GSH) levels were analyzed in sera from peripheral blood of ALS patients and age-matched control subjects at two visits separated by 6 months. Mainly, significant increases were noted in levels of IL-6 and IL-8 cytokines; IL-1β level was also elevated in sera from SALS patients vs. controls. Also, significantly reduced GSH and elevated nitrite levels were detected in ALS patients in both visits, indicating ongoing oxidative stress likely due to an imbalance caused by excessive generation of pro-oxidants and insufficient anti-oxidant mechanisms [68,69]. However, a significant increase of IL-6 in sera was determined in ALS patients vs. controls at first visit and a drastic reduction of this cytokine to control levels was noted at second visit. Our findings [45], at least, on initial high IL-6 level are supported by a previous report [44] showing increased IL-6 cytokine levels in sera from ALS patients in correlation with disease duration in range of 0.5–3 years. Though, data on IL-6 concentrations was analyzed from a small cohort ALS patients (*n* = 11), from which undetectable cytokine levels were noted in three patients. Additionally, significantly higher IL-6 levels were found in cerebrospinal fluid from ALS patients than patients with other neurological diseases [70]. However, discrepancies in IL-6 concentrations between two visits of ALS patients in our study [30] may indicate initial humoral inflammatory status and later infiltration of this protein into CNS tissues. Of note, IL-6 can cross the blood-brain barrier [71]. Permeation of IL-6 in addition to extravasation of immune/inflammatory cells (neutrophils, activated monocytes, and T lymphocytes) expressing this cytokine potentially escalate CNS inflammatory response in ALS. However, particular mechanisms of composed IL-6 actions on motor neuron function need to be explored since neurons might also be a source of IL-6 production in the brain [72]. Furthermore, it has been shown that IL-6 increases may be related to the hypoxia experienced by some ALS patients and may not be indicative of inflammatory status [73].

Our study [45] showed not only significant increases of IL-6, but also in IL-8. Although IL-8 levels were elevated in sera of ALS patients at the first visit, a significant decrease of this protein was determined 6 months later. IL-8 is a member of the Cysteine-X-Cysteine (CXC) chemokine family. IL-8 is produced by various cells, including macrophages and endothelial cells [74] and is mainly known as a pro-inflammatory mediator [75]. Increased HLA-DR (a MHC class II cell surface receptor) expression on monocytes and macrophages has been shown in SALS patient blood during disease progression, suggesting a correlation between systemic macrophage activation and ongoing CNS pathogenic processes [63]. Stimulation of human umbilical vein endothelial cells (HUVECs) with IL-8 in vitro increased endothelial cell permeability by downregulation of tight junction proteins [76]. Yet, recombinant human IL-8 enhanced HUVEC survival and proliferation in vitro, inhibited cell apoptosis, induced matrix metalloproteinase (MMP) production, and regulated angiogenesis [77]. 

Thus, the elevated pro-inflammatory cytokines and other factors found in blood of ALS patients may be imperative mediators inducing inflammatory EC response. Specifically, the IL-6 cytokine is a possible early extrinsic effector leading to EC inflammation and eventual cell degeneration in ALS.

## 3. IL-6 Cytokine and Its Receptors

The cytokine IL-6 is a multifunctional protein for regulation of metabolic and regenerative cell processes and is secreted by various cells, including ECs. This cytokine is expressed by unstimulated neutrophils and eosinophils in peripheral blood of healthy donors at variable levels [78]. This suggests active contribution of granulocytes to the IL-6 cytokine content in the systemic compartment. It has been shown that elevated expression of both IL-6 mRNA and protein level in unstimulated neutrophils depend on binding affinity of the constant portion of immunoglobulin G (IgG) to cell surface Fc receptors [79], leading to initiation of neutrophil activation. Blood-borne or blood-derived granulocyte IL-6 production can be rapidly up-regulated with granulocyte-macrophage colony-stimulating factor (GM-CSF), granulocyte colony-stimulating factor (G-CSF), or TNF-α [79,80]. Upon these specific stimuli, initiation of humoral or cellular immune responses may lead to a transition from innate to adaptive immunity. IL-6 is produced by T and B lymphocytes [81] and this cytokine even induced B cell maturation to augment antibody production [82] as well as growth of T cells and differentiation of naïve CD4+ T cells [83,84]. However, production of cytokines by B lymphocytes, in contrast to T cells, depends on B cells activation and differentiation state to plasma cells. Interestingly, it has been shown that IL-6 expressed by B cells upon initiation of immunoglobulin production and TNF-α secretion is associated mainly with cell proliferation [85]. Vazquez et al. [86] comprehensively discussed the role of several cytokines such as interleukins (IL-7, IL-4, IL-6, and IL-10) and interferons (IFN-α, IFN-β, and IFN-γ) on B cell development, survival, differentiation, and proliferation in regulation of antibody-mediating humoral immunity. Based on evidence that cytokine production by B cells is reliant not only on receiving activation stimuli but also on specific immune microenvironment, the authors conclude that B cells are “regulatory cells of the immune system” and “should be considered an integral component of the adaptive immune system” [86].

Peripheral monocytes are also a cellular producer of cytokines, including IL-6 (reviewed in [87]). Upon activation of monocytes and differentiation into macrophages, these cells have protective effect by phagocytizing various foreign substances and pathogens [88,89]. There are three distinct human monocyte subsets based on relative surface expression of co-receptors CD14 and CD16: classical (CD14++/CD16−), non-classical (CD14+/CD16++), and intermediate or transitional (CD14++/CD16+) [90,91,92]. These heterogenic monocyte phenotypes are closely related to their functions [93,94]. Mukherjee et al. [95] reported that “classical” monocytes are primarily phagocytic cells producing IL-10 with no inflammatory actions; “non-classical” monocytes exhibit inflammatory characteristics upon their activation by producing TNF-α and IL-1β and might also act as antigen presenting cells; “intermediate” monocytes, as a minor transitional cell subset, show both phagocytic and inflammatory function. Also, circulating blood monocytes are capable of migrating to an inflamed tissue site, including the CNS, providing defensive effect by phagocytizing host cell debris to lower inflammation and then might exacerbate inflammation by producing excessive amounts of pro-inflammatory cytokines such as TNF-α, IL-1, IL-6, IL-8, and IL-12 [87,96]. Interestingly, tissue-resident macrophages initially promote neutrophil infiltration into inflamed tissue sites followed by extravasation of inflammatory monocytes (reviewed in [96]). During this process, as the authors discussed, neutrophils produce soluble complexes of IL-6 and it receptors, activating endothelial cells to express C-C motif chemokine ligand 2 (CCL2) and vascular cell-adhesion molecule 1 (VCAM1). Extravasation of leukocytes into the CNS tissue is a multistep process: rolling, activation, arrest, crawling, and migration across the endothelial cells via the paracellular or transcellular pathway (reviewed in [97]). Each step of transendothelial cell migration is tightly controlled at the molecular level. For example, adhesion molecules (E-, P-, and L-selectins) mediate leukocyte rolling on activated endothelia in an initial step in the recruitment of leukocytes to an inflammatory site [98]. When human peripheral blood monocytes were cultured in P-selectin-coated plates, the secretion of IL-1β, IL-6, IL-8, IL-12, and macrophage inflammatory protein MIP-1β by monocytes was 10-fold higher compared with unstimulated monocytes after 20 h of culture [99]. Results of this study demonstrated that P-selectin has an important role in monocyte trafficking and cytokine production by these cells. 

Additionally, endothelial cells per se have an important role in inflammation by responding to endogenous and exogenous pro-inflammatory stimuli by production of various cytokines, chemokines, and adhesion molecules. In one study [100], HUVEC treated with pro-inflammatory factors such as lipopolysaccharide (LPS), TNF-α, or IL-1β in vitro showed different cell responses to stimuli. High expression of IL-6, IL-8, and E-selectin in cell supernatants was mainly determined by IL-1β induction vs. LPS or TNF-α, leading to the authors’ suggestion that this cytokine has an imperative role in neutrophil recruitment through endothelial cells. However, another early study [101] showed that LPS, TNF, and IL-1α rapidly enhance active IL-6 production by HUVEC in addition to affecting cell proliferation. A different study [102] demonstrated that CD16+, not CD16−, a subset of peripheral blood monocytes producing high levels of IL-6, chemokine CCL2, and MMP-9 upon being cultured with TNF/IFN-γ activated HUVEC expressing CX3CL1 (C-X3-C motif chemokine ligand 1). These results suggest that interaction of CD16+ monocytes with CX3CL1-expressing endothelial cells leads to monocyte extravasation. Also, endothelin, a potent vasoconstrictor hormone produced by endothelial cells, stimulated expression of IL-6 by a rat aortic endothelial cell clone inducing endothelium inflammation [103]. Although results of these studies have important scientific value to determine specific inflammatory inducers in endothelial cell response, combination of various cytokines and other factors should be taken into account to better mimic the microenvironment to which endothelial cells might be exposed. Likewise, using endothelial cells damaged or degenerated due to different diseases might be a useful tool for understanding mechanisms of cell damage.

Additionally, the IL-6 cytokine was found to be produced by muscle contraction and released into the blood, mediating anti-inflammatory effects both systemically and locally in the muscle itself [104]. This muscle-derived IL-6, known as a “myokine” or “exercise factor”, mainly suppressed pro-inflammatory cytokine TNF-α production, induced lipolysis, stimulated cortisol expression, and enhanced muscle glycogen levels [105,106]. Production of the IL-6 myokine during exercise also stimulated anti-inflammatory cytokine IL-1 receptor agonist (IL-1ra) and IL-10 appearance in circulating blood [107,108]. The anti-inflammatory effect of exercise by production of IL-6 myokine has important benefits not only for muscle homeostasis, but also for general health. However, contradictory study results of IL-6 myokine in metabolic regulation or even in muscle function per se, indicating a pleiotropic protein effect, were noted and comprehensively reviewed [109,110]. Also, distinguishing muscle-derived IL-6 from blood-borne IL-6 in the systemic compartment might be difficult.

Together, IL-6 cytokine actions are complex and potentially dependent on the levels of other humoral cytokines, which may regulate peripheral cell-cell interaction and function. Peripheral cell cross-talk might rely not only on production of specific cytokines, but also on cytokine composition and the cytokine network within circulating blood microenvironment under physiological or pathological conditions. For example, anti-inflammatory IL-10 cytokine represses the expression of TNF-α, IL-6, and IL-1 cytokines by activated microphages whereas TGF-β counteracts IL-6′s inflammatory effects (reviewed in [111]). However, the role of IL-6 as a primary or secondary inducer in cell response due to interaction with other cytokines is still unclear. 

IL-6 can act as an anti- or pro-inflammatory mediator [49,50,51,52]. Numerous comprehensive reviews [112,113,114,115,116,117] discuss mediation of anti-inflammatory functions of IL-6 by the classic signaling pathway [112,113], whereas pro-inflammatory IL-6 responses are facilitated via the trans-signaling pathway [113,115,117]. In the classic signaling pathway, IL-6 stimulates target cells via binding membrane receptor IL-6R in association with the signaling receptor glycoprotein 130 (gp130) initiating *intracellular* signaling by activation of JAK and other signal transduction molecules (MAPK, ERK, P13K, STAT) [112,114,115]. However, IL-6R can be released by proteolytic shedding from neutrophils or by secretion from monocytes of an alternatively spliced messenger RNA (mRNA) species as a soluble form (sIL-6R) that can bind IL-6 and form binary IL-6/sIL-6R complex in sera [118,119,120,121]. This complex then binds to gp130 on cell membranes, triggering the *intracellular* trans-signaling pathway [113,122]. Using this trans-signaling mechanism, IL-6 is able to stimulate cells that lack the endogenous membrane IL-6R. Importantly, sIL-6R, which comprises the *extracellular* portion of the receptor, binds IL-6 with a similar affinity as the membrane bound IL-6R. 

High levels of IL-6 and sIL-6R have been demonstrated in several chronic inflammatory and autoimmune diseases [123,124], suggesting IL-6/sIL-6R complex involvement in the transition from acute to chronic inflammation [125]. It has been shown that IL-6/sIL-6R complex induces a pro-inflammatory response in ECs that express gp130, but not IL-6R [122,126,127]. Also, adding IL-6 to cultured bovine vascular endothelial cells for 21 h substantially increased endothelial permeability by rearranging actin filaments and by changing endothelial cell morphology [128]. Thrombin-activated HUVECs secreted IL-6 in vitro and added exogenous sIL-6R, leading to significantly increased IL-6 and monocyte chemotactic protein-1 (MCP-1) productions [127]. These effects were blocked by anti-IL-6 or anti-sIL-6R monoclonal antibodies, suppressing formation of IL-6/sIL-6R/gp130 complex. Moreover, blockage of IL-6 trans-signaling actions in the brain of bigenic GFAP-IL-6/sgp130 mice significantly reduced vascular changes and BBB leakage in addition to decreasing gliosis and enhancing hippocampal neurogenesis, suggesting that sgp130 blocks trans-signaling thereby lessening detrimental effects of IL-6 in the CNS [129]. In the phase III clinical trial, inhibition of IL-6R with tocilizumab significantly improved disease outcomes in patients with rheumatoid arthritis [130]. 

However, recent studies demonstrated that IL-6 deficiency does not affect disease outcomes in G93A SOD1 mice modeling ALS [131] and IL-6 blockage with a murine surrogate of tocilizumab revealed deleterious clinical effects in these animals despite a modest anti-inflammatory impact [132]. However, the role of IL-6 pathways upon EC status in ALS is not a focus of these important studies. 

## 4. Effects of ALS Plasma Proteins on Human Bone Marrow Derived Endothelial Cells In Vitro

Plasma samples were obtained from clinically definite sporadic ALS patients and healthy controls during peripheral blood processing for mononuclear cell isolation as described [45,133]. This study was approved by the Institutional Review Board at the University of South Florida (IRB #103861, 31 May 2007). Each participant in the study signed an informed consent form prior to enrollment. Plasma samples from a randomly selected SALS male patient at moderate disease stage (ID #004, Amyotrophic Lateral Sclerosis Functional Rating Scale-Revised (ALSRS-R) 22, 69 years old) and an age-matched male control subject (ID #AB, ALSRS-R 48, 65 years old) were used for our preliminary in vitro studies.

The effect of plasma proteins from the ALS patient and control subject on EC homeostasis and on IL-6R and occludin (tight junction protein) immunoexpressions in ECs was determined using human bone marrow derived endothelial cells (hBMECs, CELPROGEN Inc., Torrance, CA, USA). Initially, hBMECs plated at a density of 5 × 10^4^/mL into 24-well plates in Celprogen Complete Growth Media for 48 h developed a cobblestone morphology and tubular vessel formation. When 10% plasma from the ALS patient was added to culture media, hBMECs demonstrated a disorganized morphology as characterized by swelling, formation of numerous cytoplasmic vesicles, and reduction of cell processes after 48 h. The cells, after adding plasma from the control patient, also displayed cytoplasmic vesicles but morphology such as tubule formation was similar to control cells. These results were supported by our previous report [31] that demonstrated swollen and vacuolated endothelial cells in capillaries of the medulla, cervical, and lumbar spinal cord of post-mortem tissues from SALS. 

Follow-up studies were performed using a sensitive BBB in vitro model as described [134]. This BBB model, composed only of hBMECs, allows defining particular molecular mechanism(s) that affect ECs by ALS humoral effectors without influence/interaction from other cellular BBB components. Briefly, a monolayer of hBMECs (10^5^ cells/200 µL) was plated onto a culture insert with semi-permeable membrane (1 µm) in Celprogen Complete Growth Media for 24 h. Then, 10% FBS, plasma from ALS or control patient was added to culture media into an insert compartment. Cells were fixed on a membrane after 5 days in vitro (DIV) and double immunohistochemical staining was performed for IL-6R and occludin using IL-6R mouse monoclonal antibody and rabbit polyclonal anti-occludin antibody, respectively. Then cells were incubated with appropriate secondary antibody conjugated to FITC or rhodamine. Fluorescence immunoexpressions of IL-6R and occludin were analyzed in obtained immunohistochemical images by measuring integrated density of positive cell expression per area using ImageJ software (National Institutes of Health, Bethesda, MD, USA, https://imagej.nih.gov/ij/, version 1.46). 

**Figure 1:**
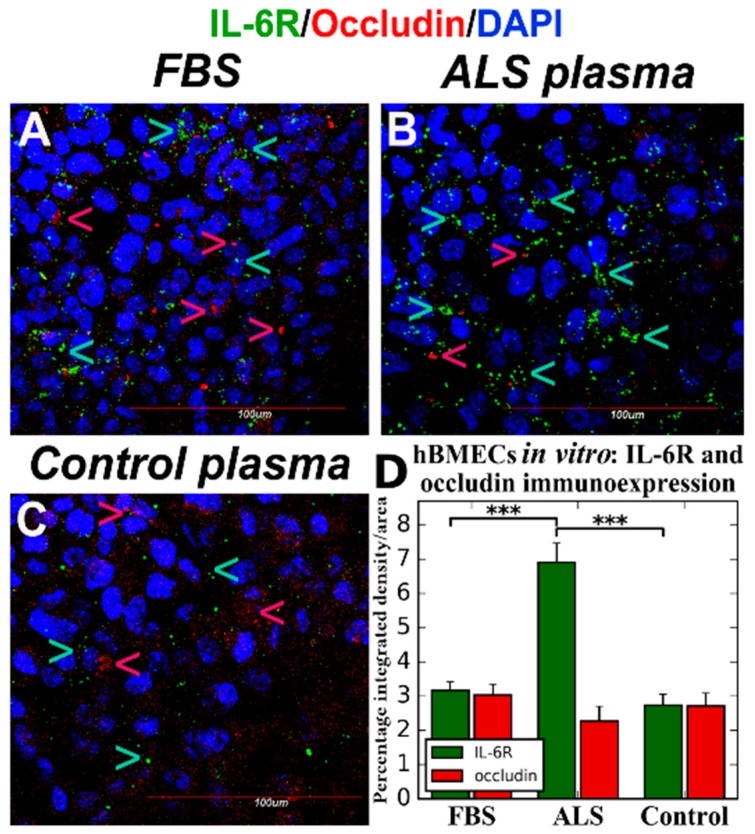
Confocal fluorescent images of hBMECs immunostained for IL-6R and occludin in vitro. Double immunostaining for IL-6R and occludin was performed on fixed hBMECs after 5 DIVexposure to FBS, plasma from ALS or control patient. The cells containing ALS plasma in media demonstrated significantly increased IL-6R (green, arrow) and reduced occludin (red, arrow) immunoexpressions (**B**,**D**). There were no differences in IL-6R or occludin immunoexpression between culture cells after exposure to FBS (**A**) or plasma from control subject (**C**). DAPI (blue) was used for nuclei staining. Data are presented as means ± S.E.M. Scale bar in (**A**–**C**) is 100 µm. *** *p* < 0.001.

Results showed a significant (*p* < 0.001) increase of IL-6R immunoexpression in ECs by exposure to ALS plasma (Figure 1B,D) vs. plasma from control patient (Figure 1C,D) or FBS (Figure 1A,D). Also, occludin immunostaining displayed a tendency towards downregulation after adding plasma from an ALS patient (Figure 1D).

**Figure 2:**
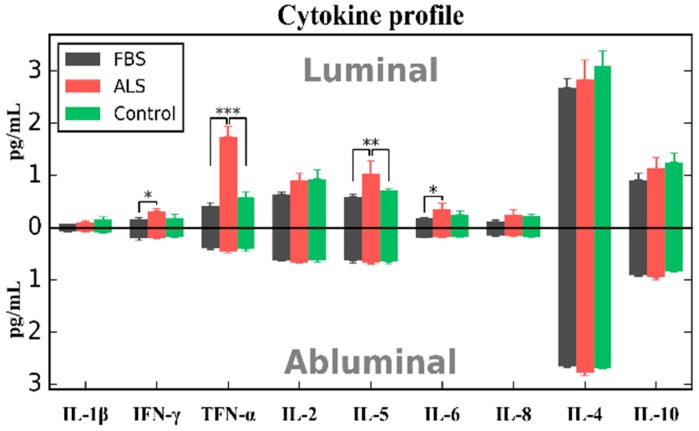
Cytokine profile of media collected from luminal (**upper**) and abluminal (**lower**) compartments. Media was collected from luminal (insert, upper) and abluminal (24-well plate, lower) compartments separated by a porous membrane at 5 DIV after exposure to FBS, plasma from ALS or control patient. At luminal side, significant increase of IFN-γ, TNF-α, IL-5, and IL-6 cytokine concentrations were determined at luminal compartment primarily after cell exposure to ALS plasma. There were no differences in cytokine levels between any cell culture conditions at abluminal side of construct. Data are presented as means ± S.E.M. * *p* < 0.05, ** *p* < 0.01, *** *p* < 0.001.

Additionally, cytokine profile (IL-1β, IFN-γ, IL-2, IL-4, IL-5, IL-6, IL-8, IL-10, and TNF-α) using multiplex cytokine assay (Thermo Fisher Scientific, Waltham, MA, USA) as described [30] was performed in collected media supernatant from insert (luminal) and 24-well plate (abluminal) compartments prior to hBMEC fixation after 5 DIV of exposure to 10% FBS, plasma from ALS or control patient. Our preliminary data showed significantly elevated concentrations of IFN-γ (*p* < 0.05), TNF-α (*p* < 0.001), IL-5 (*p* < 0.01), and IL-6 (*p* < 0.05) cytokines at luminal (insert) compartment primarily after cell exposure to ALS plasma (Figure 2). However, significant difference in IFN-γ and IL-6 concentrations was shown only between ALS plasma and FBS exposures. Interestingly, no differences (*p* > 0.05) in cytokine levels were found between any cell culture conditions at abluminal (24-well plate) side. 

Thus, our preliminary in vitro study results suggest that short term exposure to plasma from ALS patient leads to morphological changes in cultured hBMECs, promotes significant IL-6R immunoexpression, and induces pro-inflammatory cell response. However, since ECs do not express IL-6R [122], detected high expression of these receptors likely reflects cellular IL-6/sIL-6R/gp130 complex formations inducing pro-inflammatory IL-6 trans-signaling, a possibility we are currently investigating. Hypothesizing that humoral IL-6-mediated inflammation triggers EC damage, then restoration of EC integrity with an anti-inflammatory agent(s) preventing IL-6/sIL-6R complex formation or inhibiting membrane trans-signaling sIL-6R/gp130, may afford vascular repair in ALS.

## 5. Proposed Mechanisms of Humoral IL-6-Mediated Inflammation Triggering Endothelial Cell Damage

We hypothesize that humoral IL-6-mediated inflammation is an early extrinsic effector leading to EC degeneration that principally contributes to B-CNS-B damage in ALS.

**Figure 3:**
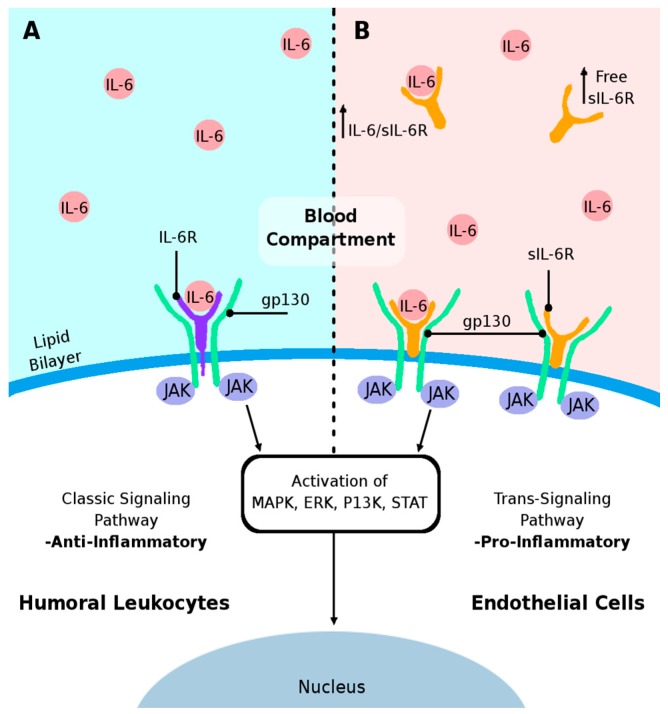
Schematic diagram proposed mechanism of humoral IL-6-mediated inflammation in triggering EC damage. (**A**) The IL-6 cytokine is an important anti-inflammatory protein for regulation of cell survival by binding to the cell membrane receptor IL-6R/gp130 complex leading to activation of JAK, subsequent activation of other signal transduction molecules which influences nuclear gene transcription (down arrow) via the classic signaling pathway; (**B**) Excessive sIL-6R (up arrow) in the blood, resultant of cleavage from the membrane (shedding) or de novo synthesis, could bind to excess IL-6 and form IL-6/sIL-6R/gp130 complex on ECs. This complex formation on the EC membrane would result in the activation of signal transduction kinases and induce a pro-inflammatory response by activating the trans-signaling pathway.

Cytokine IL-6 is a multipotent protein with anti-inflammatory and pro-inflammatory effects. As discussed above, the anti-inflammatory action of IL-6 promotes physiological cell function by binding to the cell membrane receptor IL-6R/gp130 complex via the classic signaling pathway [112,113,114]. In the systemic compartment, leukocyte function might be regulated by various classic intracellular transduction molecules mediating immune/inflammatory cell response in both innate and adaptive immunity (Figure 3A). Elevated IL-6 cytokine levels in blood allow binding of IL-6 to the soluble receptor sIL-6R leading to IL-6/sIL-6R complex formation [118,119,120,121,135]. Upon binding of this complex to gp130 on the cell membrane, a pro-inflammatory cell response is induced by activation of the trans-signaling pathway [113,115] (Figure 3B). Of note, endogenous regulatory mechanisms such as a soluble form of gp130 (sgp130) have been detected in human blood [136,137]. This isoform of sgp130 can bind to the IL-6/sIL-6R complex in the blood circulation and may specifically inhibit IL-6-mediated trans-signaling [113,115].

Accumulated evidence indicates that the humoral immune/inflammatory system response is highly involved in ALS pathogenesis. Potential increase of free sIL-6R is the result of shedding from various humoral cells with primary cellular contributors being neutrophils and activated monocytes. Elevated levels of free sIL-6R promote formation of the IL-6/sIL-6R complex in blood following binding to gp130 on target cell membranes, which activates the trans-signaling pathway and potential induction of pro-inflammatory response in EC. Additionally, activation of this trans-signaling pathway in ECs promotes the de novo synthesis of monocyte-attracting chemokines and vascular cell-adhesion molecules, leading to extravasation of inflammatory or immune cells into the CNS. However, the possibility that the IL-6/sIL-6R-mediated intracellular trans-signaling pathway may induce an EC pro-inflammatory response in ALS warrants investigation.

## 6. Conclusions and Perspectives

ALS is a complicated incurable disease with multiple etiologies and limited therapies. Numerous factors have been shown to be involved in ALS pathogenesis. One possible effector is blood-CNS barrier impairment, primarily through endothelial cell (EC) degeneration. Although mechanisms of EC damage in ALS remain unidentified, humoral inflammatory factors may initiate disease-related vascular changes. Systemic IL-6-mediated inflammation may be an early extrinsic effector leading to EC death damaging the CNS barrier. Our and other studies showed elevated levels of various inflammatory cytokines, including IL-6, in blood of ALS patients. Excessive humoral IL-6 cytokine levels could induce a pro-inflammatory EC response by activating the trans-signaling pathway, as discussed in this review.

We initiated studies to determine the effect of plasma proteins from ALS patients on EC homeostasis. Our preliminary in vitro studies demonstrated a proof of principle that short term exposure of hBMECs to plasma from ALS patient deteriorates cell morphology and promotes significant IL-6R immunoexpressions, likely reflecting cellular IL-6/sIL-6R/gp130 complex formations. Also, occludin immunostaining displayed a tendency towards downregulation. It is possible that significantly reduced “tightness” between ECs in vitro and increased endothelial permeability could be determined after long term ALS plasma exposure. Additionally, adding ALS plasma at luminal (insert) compartment using BBB in vitro model led to significantly increased IFN-γ, TNF-α, IL-5, and IL-6 cytokine concentrations. These results suggest an induced pro-inflammatory EC response by secretion of specific factors even after brief exposure to humoral proteins from the ALS patient. Yet, cytosolic EC content should confirm inflammatory cell status. Together, our preliminary data showed that EC dysfunction in ALS is potentially initiated by the detrimental humoral inflammatory modulator IL-6 cytokine.

Following our initial study results, our research team will continue to test the hypothesis of humoral IL-6-mediated inflammation triggering EC damage towards the goal of identifying pathogenic mechanism(s) in ALS vascular impairment. For instance, ALS plasma samples obtained at different disease stages and/or increased exposure of ECs in vitro to ALS plasma may be essential to mimic clinical outcomes on EC damage. Since IL-6 induces the trans-signaling inflammatory pathway via soluble receptors in pathophysiological situation, exploring molecular mechanisms of this pathway is important to understand inflammatory EC response in ALS. Primarily, determining free sIL-6R levels and IL-6/sIL-6R complexes in plasma from ALS patients with initial, moderate, and advanced disease stages is imperative to confirm the role of the IL-6 cytokine as a mediator of EC inflammation. Also, membrane trans-signaling sIL-6R/gp130 complex formations on ECs should be resolved after exposure of ALS plasma proteins. Additionally, investigation of the downstream *intracellular* signaling pathway by involvement of JAK or other signal transduction molecules may be essential to determine EC alterations in ALS. 

We are currently performing the above mentioned and other studies, which may form the basis for a therapeutic approach towards vascular repair via an endothelial-IL-6-targeting therapy in ALS. An anti-inflammatory agent for EC restoration could be approached for modulating IL-6 induced inflammation by decreasing sIL-6R levels and IL-6/sIL-6R complex formations or by inhibiting membrane trans-signaling sIL-6R/gp130 in EC membrane. Also, modulation of soluble IL-6R rather than the membrane-bound IL-6R may be a promising approach to regulate and/or control pro-inflammatory effect of IL-6 via suppression of IL-6 trans-signaling activation, leading to EC repair in ALS.

## Abbreviations

ALS	amyotrophic lateral sclerosis
ALSRS-R	amyotrophic lateral sclerosis functional rating scale-revised
BBB	blood-brain barrier
B-CNS-B	blood-CNS barrier
BSCB	blood-spinal cord barrier
CCL2	C-C motif chemokine ligand 2
CNS	central nervous system
CX3CL1	C-X3-C motif chemokine ligand 1
CXC	cysteine-X-cysteine
DAPI	4′,6-diamidino-2-phenylindole
DIV	days in vitro
EC	endothelial cell
ERK	extracellular signal-regulated kinase
FALS	familial ALS
FBS	fetal bovine serum
FITC	fluorescein Isothiocyanate
G-CSF	granulocyte colony-stimulating factor
GM-CSF	granulocyte-macrophage colony-stimulating factor
gp130	glycoprotein 130
GSH	glutathione
hBMEC	human bone marrow derived endothelial cell
hUVEC	human umbilical vein endothelial cell
IFN-β	interferon-beta
IFN-γ	interferon-gamma
IgG	immunoglobulin G
IL	interleukin
IL-6R	interleukin-6 receptor
JAK	janus kinase
LPS	lipopolysaccharide
MAPK	mitogen-activated protein kinase
MCP-1	monocyte chemotactic protein-1
MHC	major histocompatibility complex
MMP	matrix metalloproteinase
mRNA	messenger RNA
PI3K	phosphatidyl-inositol-3-kinase
SALS	sporadic ALS
sgp130	soluble form of gp130
sIL-6R	soluble form of IL-6R
SOD-1	superoxide dismutase 1
STAT	signal transducer and activator of transcription
TGF- β	transforming growth factor-beta
TNF-α	tumor necrosis factor-alpha
